# Association of Staffing Instability With Quality of Nursing Home Care

**DOI:** 10.1001/jamanetworkopen.2022.50389

**Published:** 2023-01-10

**Authors:** Dana B. Mukamel, Debra Saliba, Heather Ladd, R. Tamara Konetzka

**Affiliations:** 1 Public Health and Nursing, information Technology Enhancing Quality Care (iTEQC) Research Program, Division of General Internal Medicine, Department of Medicine, University of California, Irvine, Irvine; 2 University of California, Los Angeles Borun Center at David Geffen School of Medicine, Los Angeles; 3 Geriatric Research, Education, and Clinical Centers, Veterans Administration, Los Angeles, California; 4 RAND, Santa Monica, California; 5 iTEQC Research Program, Division of General Internal Medicine, Department of Medicine, University of California, Irvine, Irvine; 6 Department of Public Health Sciences, The University of Chicago, Chicago, Illinois

## Abstract

**Importance:**

Recent work suggests that instability in nursing home staffing levels may be an important marker of nursing home quality. Whether that association holds when controlling for average staffing levels is unknown.

**Objective:**

To examine whether staffing instability, defined as the percentage of days below average staffing levels, is associated with nursing home quality when controlling for average staffing levels.

**Design, Setting, and Participants:**

This quality improvement study of 14 717 nursing homes used the merged Centers for Medicare & Medicaid Services Payroll Based Journal, Minimum Data Set, Nursing Home Care Compare, and Long-Term-Care Focus data for fiscal years 2017 to 2019. Statistical analysis was performed from February 8 to November 14, 2022.

**Main Outcomes and Measures:**

Linear, random-effect models with state fixed effects and robust SEs were estimated for 12 quality indicators as dependent variables, percentage of below-average staffing days as independent variables, controlling for average staffing hours per resident-day for registered nurses, licensed practical nurses, and certified nurse aides. Below-average staffing days were defined as those 20% below the facility average, by staffing type. Quality indicators included deficiency citations; long-stay residents receiving an antipsychotic; percentage of high-risk long-stay residents with pressure ulcers (2 different measures for pressure ulcers were used); and percentage of long-stay residents with activities of daily living decline, mobility decline, emergency department visits, and hospitalizations; and short-stay residents with new antipsychotic medication, mobility decline, emergency department visits, and rehospitalizations.

**Results:**

For the 14 717 nursing homes in this study, the mean (SD) percentage of days with below-average staffing was 30.2% (12.0%) for registered nurses, 16.4% (11.3%) for licensed practical nurses, and 5.1% (5.3%) for certified nurse aides. Mean (SD) staffing hours per resident-day were 0.44 (0.40) for registered nurses, 0.80 (0.32) for licensed practical nurses, and 2.20 (0.50) for certified nurse aides. In regression models that included average staffing, a higher percentage of below-average staffing days was significantly associated with worse quality for licensed practical nurses in 10 of 12 models, with the largest association for decline of activities of daily living among long-stay residents (regression coefficient, 0.020; *P* < .001). A higher percentage of below-average staffing days was significantly associated with worse quality for certified nurse aides in 9 of 12 models, with the largest association for short-stay functioning (regression coefficient, 0.030; *P* = .01).

**Conclusions and Relevance:**

This study suggests that holding average staffing levels constant, day-to-day staffing stability, especially avoiding days with low staffing of licensed practical nurses and certified nurse aides, is a marker of better quality of nursing homes. Future research should investigate the causes and potential solutions for instability in staffing in all facilities, including those that may appear well-staffed on average.

## Key Points

**Question:**

Is day-to-day staffing instability of registered nurses, licensed practical nurses, and certified nurse aides, measured as the percentage of days substantially below average levels, associated with nursing home quality?

**Findings:**

In this quality improvement study of 14 717 nursing homes, after controlling for average staffing levels, staffing instability of licensed practical nurses and certified nurse aides was associated with lower quality across standard quality measures.

**Meaning:**

This study suggests that day-to-day staffing instability is an important marker of nursing home quality, limiting measurement, reporting, or policy to average hours per resident-day likely will miss important opportunities to improve care.

## Teaser

This quality improvement study of 14 717 nursing homes examines whether staffing instability, defined as the percentage of days below average staffing levels, is associated with nursing home quality when controlling for average staffing levels.

## Introduction

The association between staffing levels and quality of nursing home care has been a topic of research, policy debates, and federal and state regulations for decades. Recently, the White House announced that, within a year the federal government will implement a new minimum staffing policy for nursing homes.^1^ The evidence linking staffing levels to quality of care is substantial.^2,3,4,5,6,7,8,9^ Several studies found that higher staffing levels (more registered nurses [RNs] and certified nurse aides [CNAs], and, in fewer studies, also more licensed practical nurses [LPNs]) led to fewer deficiency citations for deviations from state and federal quality standards,^10^ fewer hospitalizations and emergency department (ED) visits, and better scores on other quality measures (QMs), including some of those reported in the Nursing Home Care Compare (NHCC)^11^ report card published by the Centers for Medicare & Medicaid Services (CMS).

This evidence base and NHCC rely on measuring staffing as average levels over time. A recent study has shown that when staffing variations are examined on a daily basis, different patterns emerge, revealing additional information about the association between nurse staffing levels and quality of care. Mukamel et al^12^ defined new measures of below-average staffing days as the number of days during a period (eg, 1 year) in which the number of RN (or CNA or LPN) staffing hours per resident-day (HPRD) is less than 20% of the facility’s average for the period. They have shown, for a national sample of nursing homes, that these measures are associated with other QMs such as the Five-Star QMs and survey ratings published by the CMS, as well as facility ownership. That study was the first to introduce the concept that quality of care may depend not only on the average level of staffing but also on staffing stability over time, a concept common in industrial quality improvement,^13^ but infrequently applied when reporting on factors associated with health care outcomes and quality. The study demonstrated the importance and relevance of this concept in producing high-quality care in nursing homes.^12^

In the present study we add to this foundational work that established a working definition of staffing instability by rigorously testing the hypothesis that staffing instability adds information about quality of care above and beyond the traditional average staffing measure. We estimate 12 regression models, 1 for each of 12 standard QMs, with all models including both the instability and the average staffing measures for RNs, LPNs, and CNAs. These models allow us to test the hypothesis that instability in any of these of staffing measures is associated with lower quality of care as measured by these QMs, while controlling for average staffing levels as well as other nursing home characteristics.

## Methods

### Data and Sample

Of the 15 790 nursing homes submitting staffing data to the CMS Payroll Based Journal during fiscal years 2017-2019, we excluded those missing independent or dependent variables. Because we estimated separate models for each of the dependent variables, sample sizes varied from 10 037 to 14 183. A total of 14 717 facilities (93% of the Payroll Based Journal sample) appeared in at least 1 model. This study followed the Standards for Quality Improvement Reporting Excellence (SQUIRE) reporting guideline.^14^ The study was reviewed by the University of California Irvine’s institutional review board with consent waived due to the large number of individuals whose data were included in the Minimum Data Set, making it infeasible to obtain consent. The data were obtained deidentified under a data use agreement from CMS.

We created an analytical data set by linking data sets at the nursing home level using the CMS Provider Number of the nursing home. Daily hours worked by direct-care nurses by type (RNs, LPNs, and CNAs) as well as daily resident census were obtained from the Payroll Based Journal, which is the source of staffing data for NHCC. Quality measures, obtained from NHCC,^15^ are published and updated regularly by CMS for all nursing homes nationally. Nursing home organizational characteristics were obtained from the Long-Term-Care Focus website.^16^ Nursing home characteristics reflecting characteristics of each resident in the facility were obtained from the CMS Minimum Data Set, version 3. These data are submitted to the CMS by all Medicare- and Medicaid-certified nursing homes and include assessments for all residents with information about age, sex, health status, and treatments. The CMS uses these data to calculate a case mix index for payment and the QMs published in the NHCC.

### Variable Definitions

#### Dependent Variables

The dependent variables were the QMs included in the CMS Five-Star NHCC ratings during 2017-2019 that have not topped out (ie, did not converge such that little variation across facilities remains), as follows (for all, lower values indicate better quality). (1) Health deficiency score was based on the most recent health inspection of the year. The score is calculated as the sum of points assigned to the nursing home based on the number, scope, and severity of the deficiencies.^17^ To adjust for variation across states,^18^ the scope and severity–adjusted number of deficiencies for each facility were converted to a *z* score based on the mean and SD of deficiencies in its state. (2) Long-stay QMs: percentage of residents who received an antipsychotic medication, percentage of high-risk residents with pressure ulcers (2 different measures for pressure ulcers were used), percentage of residents whose ability to move independently worsened, percentage of residents whose need for help with activities of daily living has increased, number of hospitalizations per 1000 long-stay resident-days, and number of ED visits per 1000 long-stay resident-days. (3) Short-stay QMs: percentage of residents who received antipsychotic medication for the first time, who failed to improve in their ability to move independently, who were rehospitalized after a nursing home admission, and who have had an ED visit.

The definition of the long-stay QM for pressure ulcers changed in quarter 4 of 2018 and the change affected its national average. We therefore estimated separate models for the measure that was in effect from quarter 1 of 2017 to quarter 3 of 2018 and from quarter 4 of 2018 to quarter 3 of 2019.

These QMs are defined over different time intervals and, hence, reflect quality of care provided during different time periods. Specifically, hospitalizations and ED visits are measured over 1 year (eg, 2017), short-stay outcomes are measured over 6 months (eg, January-June 2017), and long-stay outcomes are measured over 1 quarter (eg, January-March 2017). Deficiency citations are issued during an annual in-person survey of the facility and presumably reflect care provided in the recent past. Our analyses assume that deficiency citations reflect care during the 6 months preceding the survey. Therefore, the date of the 180-day period over which we defined this measure is specific to each nursing home.

#### Independent Variables

We calculated daily staffing HPRDs for RNs, LPNs, and CNAs and for each day and each staffing type. If HPRDs for the day were below 20% of the facility’s average HPRD level for the period (see periodicity definitions), the day counted toward the percentage of below-average days for that staffing type for that period. This percentage measured the instability of staffing by type during the period.

Minimum Data Set data were used to calculate average facility daily case mix of residents in terms of age, sex, and Resource Utilization Group (RUGs) IV score for each nursing home. These daily case mixes of residents were based on residents’ admission and discharge dates, and assumed that a resident’s RUGs score does not change until the next Minimum Data Set assessment, which for short-stay residents could be as short as 11 days and for long-stay residents was typically 90 days. Annual values for ownership, payer mix, occupancy, hospital-based affiliation, and chain affiliation were obtained from the Long-Term-Care Focus website. Thus, these variables provided values that were updated only annually.

#### Aligning Variables and Outcomes Periodicity Definitions

Because outcomes (dependent variables) were measured over different time periods (quarters, semi-annual, annual, and facility-specific), independent variables associated with the observed outcomes had to be defined over the same time periods. We, therefore, averaged all the daily independent variables over time periods corresponding to the time period defined for each outcome. For example, for the long-stay outcomes that were measured over 1 quarter (eg, activities of daily living), all the daily measures of staffing (HPRD and instability), average daily age of the residents, and average daily RUGs score were averaged separately for each of the 11 quarters between 2017 and quarter 3 of 2019,^19^ resulting in 11 observations for each nursing home. Similarly for the short-stay measures, which were measured over 6 months, we averaged the same independent daily variables for each 6-month period, resulting in 6 observations per nursing home.

### Statistical Analysis

#### Estimated Models

Statistical analysis was performed from February 8 to November 14, 2022. We estimated 12 separate models, 1 for each QM as a dependent variable. The unit of observation was the nursing home, with repeated observations for nursing homes and number of repetitions depending on the outcome (eg, 11 for those measured quarterly). The independent variables included the 3 instability staffing measures (ie, the below-average staffing percentages) and the 3 average staffing measures in terms of HPRD, for RNs, LPNs, and CNAs. In addition, all models controlled for mean resident sex and age, payer, RUGs score, facility characteristics, and time trend. The models were estimated as ordinary least-squares regressions with random facility effects, fixed state effects, and robust SEs.

We tested the hypothesis that the instability measures for RNs, LPNs, and CNAs offer independent information about quality, above and beyond the information offered by the HPRD staffing measures in 12 separate regressions, by assessing whether the *P* values were below the .05 threshold for significance. To account for multiple comparisons we applied the Benjamini-Hochberg adjustment.^20^ We present the Benjamini-Hochberg–adjusted *P* values.

#### Sensitivity Analyses

We tested an alternative specification for instability, defining it as total days exceeding a 20% band above and below the average staffing level, and repeated the analysis with this measure.

#### Estimated Association With Outcomes

We estimated the marginal association of decreasing instability of each of the staff types—RNs, LPNs, and CNAs—by 1 SD of the instability measure for each separately, on each of the 12 outcomes. We then calculated it as a percentage of the mean of the outcome measure. We calculated similarly the same percentage for a 1-SD increase in the average staffing measures for comparison.

## Results

Of the 14 717 nursing homes participating in the study, 70.3% were for-profit facilities, 59.0% were affiliated with a chain, and 4.1% were hospital based (Table 1). Their residents’ mean (SD) RUGs score was 1.6 (0.4), a mean (SD) of 38.8% (18.1%) were aged 85 years or older, 64.4% (11.7%) were female, 60.2% (22.6%) were Medicaid beneficiaries, and mean (SD) occupancy was 80.3% (14.3%). The QM means (SDs) ranged from 1.0% (0.6%) for number of ED visits per 1000 long-stay resident-days to 31.8% (10.9%) for percentage of short-stay residents whose independent mobility did not improve. The mean (SD) percentage of days with below-average staffing for RNs was 30.2 (12.0), for LPNs was 16.4 (11.3), and for CNAs was 5.1 (5.3), with substantial variation across nursing homes, as indicated by the SDs. The corresponding mean (SD) HPRD for RNs was 0.44 (0.40), for LPNs was 0.80 (0.32), and for CNAs was 2.20 (0.50), also exhibiting substantial variation.

**Table 1.** Descriptive Statistics

Statistic	Analysis sample		Excluded from analysis		
	No. of nursing homes	Mean (SD)	No. of nursing homes	Mean (SD)	*P* value^a^
**Quality measures** ^b^					
Health deficiency score, standardized within each state by subtracting the state mean score and dividing by the SD of the state scores	13 999	0.00 (0.81)	1052	−0.01 (0.93)	.64
Long-stay residents					
Who received an antipsychotic medication, %	14 117	14.7 (8.8)	686	14.7 (10.3)	.91
High-risk residents, %					
With pressure ulcers from quarter 1 of 2017 to quarter 3 of 2018	13 427	5.5 (3.4)	600	5.7 (4.3)	.16
With pressure ulcers from quarter 4 of 2018 to quarter 3 of 2019	12 973	7.3 (4.2)	756	8.0 (4.8)	<.001
Residents whose need for help with activities of daily living has increased, %	13 962	14.8 (5.4)	659	16.2 (6.3)	<.001
Residents whose ability to move independently worsened, %	13 671	18.0 (6.6)	614	18.9 (7.7)	.001
No. of ED visits per 1000 long-stay resident days	12 854	1.0 (0.6)	1105	1.0 (0.6)	.64
No. of hospitalizations per 1000 long-stay resident days	13 299	1.7 (0.7)	952	1.6 (0.7)	<.001
Short-stay residents					
Who received antipsychotic medication for the first time, %	11 815	1.7 (1.5)	915	1.6 (1.9)	.05
Who declined or stayed the same in their ability to move around on their own, %	10 019	31.8 (10.9)	832	33.8 (13.1)	<.001
Who were rehospitalized after a nursing home admission, %	13 196	21.5 (4.9)	1158	21.6 (5.6)	.64
Who had an outpatient ED visit, %	13 196	11.0 (4.4)	1158	10.8 (4.8)	.12
**Staffing instability** ^c^					
Below-average staffing days, %					
RNs	14 717	30.2 (12.0)	1011	31.9 (12.9)	<.001
LPNs	14 717	16.4 (11.3)	948	24.0 (14.9)	<.001
CNAs	14 717	5.1 (5.3)	1028	11.6 (11.0)	<.001
**Staffing level** ^c^					
Average hours per resident-day					
RNs	14 717	0.44 (0.40)	1073	0.95 (1.10)	<.001
LPNs	14 717	0.80 (0.32)	1073	0.96 (0.67)	<.001
CNAs	14 717	2.20 (0.50)	1073	2.5 (0.9)	<.001
**Facility characteristics** ^c^					
Case-mix index	14 717	1.6 (0.4)	1066	1.9 (0.6)	<.001
Residents, %					
<65 y	14 717	15.9 (14.7)	1066	13.6 (15.2)	<.001
65-74 y	14 717	18.4 (7.9)	1066	19.2 (9.1)	.003
75-84 y	14 717	26.9 (6.7)	1066	29.4 (9.1)	<.001
≥85 y	14 717	38.8 (18.1)	1066	37.8 (17.9)	.07
Male residents, %	14 717	35.6 (11.7)	1066	37.1 (14.1)	<.001
Female residents, %	14 717	64.4 (11.7)	1066	62.9 (14.1)	<.001
Resident census, No.^c^	14 717	87.4 (52.8)	1073	54.4 (38.3)	<.001
**Residents with payer, %** ^c^					
Medicare	14 717	13.4 (12.6)	242	22.6 (30.4)	<.001
Medicaid	14 717	60.2 (22.6)	242	50.8 (35.4)	<.001
Other	14 717	26.5 (18.3)	242	26.6 (23.3)	.96
**Nursing home occupancy, %** ^d^	14 717	80.3 (14.3)	240	72.1 (21.3)	<.001
County population in rural areas, %^d^	14 717	29.5 (30.1)	951	20.9 (25.2)	<.001
County HHI^d^	14 717	0.21 (0.25)	952	0.17 (0.22)	<.001
Nursing home, No. (%)^d^					
For profit	14 717	10 346 (70.3)	242	131 (54.1)	<.001^e^
Part of a chain	14 717	8689 (59.0)	242	109 (45.0)	<.001^e^
Hospital based	14 717	598 (4.1)	242	63 (26.0)	<.001^e^

Abbreviations: CNAs, certified nurse aides; ED, emergency department; HHI, Herfindahl-Hirschman Index; LPNs, licensed practical nurses; RNs, registered nurses.

^a^ For the χ^2^ test of H_0_: there is no association between the independent variable and the analysis sample status.

^b^ As each quality measure is defined over a different time period, all statistics are averaged over their respective time periods as follows: long-stay measures are averages of quarterly data; short-stay measures are averages of 2 quarters’ data; ED and hospitalization measures are averages of annual data; and surveys are averages of 180 days’ measures.

^c^ Average of quarterly measures.

^d^ Average of annual measures.

^e^ For the *t* test of H_0_: mean for analysis sample − mean for excluded sample ≠ 0.

Table 1 also compares the included and excluded nursing homes. Six of the 12 QMs and all facility characteristics were significantly but not meaningfully different. Staffing and instability measures differed clinically as well, with both instability and HPRD averages being higher in the excluded sample, particularly for staffing instability of LPNs and CNAs.

Table 2 summarizes our main findings. The full models are presented in the eTable in Supplement 1. Because all outcome measures are defined such that lower values indicate higher quality, positive coefficients indicate that an increase of 1 unit in the independent variable is associated with higher quality. For CNAs, we found support for the hypothesis that staffing instability (percentage of days of below-average staffing levels) is significantly associated with worse quality in 9 of the 12 tests. The largest association was with the QM of functioning failing to improve by discharge among short-stay residents (regression coefficient, 0.030; *P* = .01), followed by the QM of independent mobility worsening among long-stay residents (regression coefficient, 0.017; *P* = .006). For LPNs, we found support for the hypothesis in 10 of 12 tests. The largest associations were with ED visits among short-stay residents (regression coefficient, 0.020; *P* < .001) and decline in activities of daily living among long-stay residents (regression coefficient, 0.020; *P* < .001). For RNs, we found no significant associations with any of the measures we tested.

**Table 2.** Associations Between Below-Average Staffing Levels and Quality Measures^a^

Characteristic	Below-average staffing levels per day^b^					
	RNs, regression coefficient	*P* value^c^	LPNs, regression coefficient	*P* value^c^	CNAs, regression coefficient	*P* value^c^
Deficiency score within the past 6 mo (180 d), *z* score	0.001	.56	0.002	.006	0.006	<.001
Long-stay quality measures						
Percentage of residents receiving antipsychotic drugs for first time	0.002	.56	0.004	.21	0.012	.006
Pressure ulcers						
Quarter 1 of 2017 to quarter 3 of 2018	0.000	.83	0.006	.01	0.007	.04
Quarter 4 of 2018 to quarter 3 of 2019	−0.003	.46	0.018	<.001	0.003	.47
Decline in activities of daily living	0.001	.83	0.020	<.001	0.018	.002
Percentage of residents whose ability to move independently worsened	0.001	.83	0.018	<.001	0.017	.006
ED visits per 1000 residents	0.001	.46	0.002	<.001	0.004	<.001
Hospitalizations per 1000 residents	0.000	.65	0.002	.006	0.003	.002
Short-stay quality measures						
Percentage of residents receiving antipsychotic drugs for first time	−0.000	.83	0.004	.004	0.006	.002
Functioning failed to improve by discharge	0.012	.46	0.004	.63	0.030	.01
Rehospitalizations	−0.006	.46	0.017	.003	−0.004	.54
ED visits	0.002	.83	0.020	<.001	0.006	.38

Abbreviations: CNAs, certified nurse aides; ED, emergency department; LPNs, licensed practical nurses; RNs, registered nurses.

^a^ Coefficients are from regressions controlling for average hours per resident-day for RNs, LPNs, and CNAs; residents and nursing home characteristics; state fixed effects; and time indicator variables. Because all measures are of adverse outcomes, positive values indicate decline in quality. The full regression is available in the eTable in Supplement 1.

^b^ Defined as percentage of days during the period in which staffing was 20% below the nursing home average.

^c^ Adjusted for multiple comparisons using the Benjamini-Hochberg adjustment.

Sensitivity analyses led to findings similar to those of the main analysis with respect to CNAs and LPNs, showing that total days’ outliers were also associated with lower quality of care, although the effect sizes tended to be smaller. The full regression models (eTable in Supplement 1) show similar findings to those found in prior studies with respect to average HPRDs for RNs, LPNs, and CNAs. Higher RN and CNA average staffing hours were significantly associated with better quality in most cases, while LPN staffing hours were either not significantly associated with the QMs or were associated with worse quality.^2,3,4,5,6,7,8^

Table 3 presents the estimated association of improvement with outcomes: decreasing instability and increasing average staffing measures by 1 SD for each staffing type. Because all outcomes are of adverse events, negative percentages indicate improved quality associated with the change in staffing. For RNs, only HPRD increases were associated with significant improvements, with 8 outcomes showing significant improvements, ranging from 0.18% (*P* < .001) for the deficiencies score within the past 6 months to 6.96% (*P* < .001) for ED visits per 1000 long-stay residents. For CNAs, 6 instability measures were associated with more improvement compared with HPRD, ranging from 0.05% (*P* < .001) for the deficiencies score within the past 6 months to 2.12% (*P* < .001) for ED visits per 1000 long-stay residents. The most associations of instability with outcomes were found for LPNs, with 10 outcomes associated with improvement relative to 1 for HPRD, ranging from 0.04% (*P* = .006) for the deficiencies score within the past 6 months to 2.66% (*P* = .004) for percentage of short-stay residents receiving antipsychotic drugs for the first time.

**Table 3.** Improvement in Outcomes Due to a 1-SD Change in Staffing Measures: Below-Average Days vs Average Hours per Resident-Day^a^

Characteristic	% Of mean											
	Registered nurses				Licensed practical nurses				Certified nurse aides			
	Below-average staffing days	*P* value^b^	Average HPRD	*P* value^b^	Below-average staffing days	*P* value^b^	Average HPRD	*P* value^b^	Below-average staffing days	*P* value^b^	Average HPRD	*P* value^b^
Deficiency score within the last 6 mo (180 d), *z* score	−0.02	.56	−0.18	<.001	−0.04	.006	−0.00	.84	−0.05	<.001	−0.04	.006
Long-stay quality measures												
Percentage of residents receiving antipsychotic drugs for first time	−0.16	.56	−1.90	<.001	−0.31	.21	−0.84	.03	−0.43	.006	0.39	.27
Pressure ulcers												
Quarter 1 of 2017 to quarter 3 of 2018	−0.07	.83	0.55	.68	−1.23	.01	3.08	<.001	−0.67	.05	−1.41	.03
Quarter 4 of 2018 to quarter 3 2019	0.49	.46	0.27	.80	−2.79	<.001	5.00	<.001	−0.22	.47	−2.71	<.001
Decline in activities of daily living	−0.08	.83	−2.23	<.001	−1.53	<.001	1.92	<.001	−0.64	.002	−0.21	.56
Percentage of residents whose ability to move independently worsened	−0.07	.83	−0.67	.28	−1.13	<.001	2.14	<.001	−0.50	.006	−0.73	.03
ED visits per 1000 residents	−1.20	.46	−6.96	<.001	−2.26	<.001	1.86	.02	−2.12	<.001	1.55	.03
Hospitalizations per 1000 residents	−0.28	.65	−4.38	<.001	−1.33	.006	1.26	.02	−0.94	.002	1.32	.006
Short-stay quality measures												
Percentage of residents receiving antipsychotic drugs for first time	0.21	.83	−4.94	<.001	−2.66	.004	0.58	.58	−1.87	.002	−0.21	.82
Functioning failed to improve by discharge	−0.46	.46	1.82	<.001	−0.14	.63	1.39	<.001	−0.51	.01	−1.59	<.001
Rehospitalizations	0.33	.46	−0.99	.002	−0.89	.003	1.47	<.001	0.10	.54	−0.59	.04
ED visits	−0.22	.83	−4.05	<.001	−2.05	<.001	2.08	<.001	−0.29	.38	−0.50	.28

Abbreviations: ED, emergency department; HPRD, hours per resident-day.

^a^ As the outcomes are all negative, a negative percentage change indicates improvement in the outcome. A larger negative percentage indicates larger improvement relative to the mean. For example, a 1-SD decrease in certified nurse aide and licensed practical nurse staffing instability was associated with similar improvements in deficiency citations of 0.05% and 0.04%, respectively.

^b^ Adjusted for multiple comparisons using the Benjamini-Hochberg adjustment.

## Discussion

In this study, we tested the hypothesis that staff instability, measured as percentage of below-average staffing days, is an indicator of nursing home care quality, above and beyond the traditional measure of average HPRD staffing, a measure that has been used in numerous prior studies. The below-average staffing days measure reflects the daily ability of the facility to meet the care needs of its residents. In proposing this measure, Mukamel et al^12^ hypothesized that both the average staffing level and the daily deviations from the average would be independently associated with the quality of care and with resident outcomes. The objectives of this study were to examine this hypothesis and to assess it with respect to several important outcomes reported in NHCC.

Our findings support the hypothesis proposed by Mukamel et al^12^ with respect to most outcomes we studied for CNAs and LPNs but not for RNs. We found that, for CNAs and LPNs, the traditional staffing measures, based on average HPRD, and the new measure based on percentage of days of below-average staffing, were independently associated with outcomes in regression models, where each measure serves as a control for the other. This finding indicates that each measure has a different and complementary association with the outcome, and that attempts to manage and improve quality by influencing only the average staffing level or only the daily staffing fluctuations may be associated with only partial success. A more complete understanding requires consideration of both.

The test of the hypothesis we provide in this study is rigorous, testing the independent association of the instability measures with 12 different outcomes, using an adjustment for the multiple comparisons we perform, to assess whether the measures pass the test. The CNA and LPN instability measures both show similar numbers of high associations with the outcomes, with the CNA measures being associated with 9 of the 12 QMs and the LPN measures being associated with 10 of the 12 QMs. The analysis of the relative associations with outcomes of the instability measures and the average HPRD measures mirrors this conclusion. The strongest associations of an increase of 1 SD of the instability measure compared with an increase of 1 SD in HPRD is for LPNs at 10 of the outcomes compared with 1 outcome for an increase in HPRD. For CNAs, instability is associated with more improvement in 6 of the outcomes compared with 4 of the outcomes for an increase in HPRD. Table 3 demonstrates that a 1-SD increase in HPRD also improved many of the outcomes, particularly for RNs and CNAs, a finding reported in many prior studies.^2,3,4,5,6,7,8,9^

These findings highlight the fact that both measures are important and offer different perspectives on quality of care. In particular, it seems that having enough RN hours is essential to nursing home quality, but stability of those hours does not matter as much, perhaps because nursing home managers find ways to compensate when an RN cannot show up, possibly by delegating some of those tasks to LPNs or to administrative RNs, or postponing those tasks. On the other hand, instability of LPN and CNA staffing seems to be a red flag for quality, perhaps one that consumers should know about. The ability of a nursing home to avoid days with low LPN and CNA staffing, perhaps by building more flexibility into staffing availability or better planning and anticipation of changes in staff availability or resident census, appears to offer a new pathway to quality improvement.

### Limitations

This study has some limitations. Nursing homes were excluded from our study if data on staffing or outcomes were not available, which resulted in samples of different sizes for different outcomes, primarily with missingness associated with small nursing homes, which are less likely to meet the threshold for minimum sample size for inclusion in the NHCC measures. These nursing homes tend to have higher HPRD but also higher instability. Nevertheless, the study captured 93% of nursing homes nationally in at least 1 of the estimated models. Because staffing data were available only at the facility level, all adjustments for resident risks, such as age and case mix, could be made only at the facility level.

## Conclusions

After President Biden’s recent call for setting federal minimum staffing standards for nursing homes,^1^ the CMS has announced a new study to determine the optimal level and type of nursing home staffing needs and is currently soliciting input from the public. It intends to issue proposed rules on minimum staffing requirement for nursing homes within 1 year.^21^ The information provided here, about both the HPRD and below-average staffing days, is relevant and may inform these efforts. Most important, it is clear that average staffing levels do not tell the whole story; now that the data are available to examine more nuanced aspects of staffing, such as the frequency with which a facility has below-average staffing, a more comprehensive view of nursing home staffing should be pursued.

## Notes

Supplement 1. **eTable.** Full Regression Models

Supplement 2.
Data Sharing Statement
