# Supplementary material for: Association of Staffing Instability With Quality of Nursing Home Care
Source: JAMA Netw Open. 2023 Jan 10;6(1):e2250389. doi: 10.1001/jamanetworkopen.2022.50389 (PMC9856742; doi:10.1001/jamanetworkopen.2022.50389)
Supplement: Supplement 1. — eTable. Full Regression Models [file jamanetwopen-e2250389-s001.pdf]

Supplementary Online Content

Mukamel DB, Saliba D, Ladd H, Konetzka RT. Association of staffing instability with quality of nursing home care. *JAMA Netw Open.* 2023;6(1):e2250389. doi:10.1001/jamanetworkopen.2022.50389

eTable. Full Regression Models

This supplementary material has been provided by the authors to give readers additional information about their work.

© 2023 Mukamel DB et al. *JAMA Network Open.*

**eTable.** Full Regression Models												
	**Long-stay**					**Short-stay**				**Long-stay**		
	**ADL increase**	**High-risk pressure ulcer FYs Q1 2017-Q3 2018**	**High-risk pressure ulcer FYs Q4 2018-Q3 2019**	**Anti-psychotic medi-cation**	**Mobility decline**	**Anti-psychotic medi-cation**	**Mobility did not decline**	**Re-hospitali zation**	**ED visit**	**Number of hospitali zation**	**ED visit**	**Deficien-cies: Z Score**
	**Coefficients [95% CI]**											
Percent of days Low Staffing: RN	0.001 [-0.004, 0.005]	0.000 [-0.003, 0.003]	-0.003 [-0.008, 0.001]	0.002 [-0.002, 0.006]	0.001 [-0.004, 0.007]	0.000 [-0.003, 0.002]	0.012 [-0.002, 0.026]	-0.006 [-0.014, 0.001]	0.002 [-0.005, 0.008]	0.000 [-0.000, 0.001]	0.001 [-0.000, 0.001]	0.001 [-0.000, 0.002]
Percent of days Low Staffing: LPN	0.020 [0.013, 0.027]	0.006 [0.001, 0.010]	0.018 [0.011, 0.025]	0.004 [-0.002, 0.010]	0.018 [0.010, 0.026]	0.004 [0.002, 0.007]	0.004 [-0.013, 0.022]	0.017 [0.006, 0.027]	0.020 [0.011, 0.028]	0.002 [0.001, 0.003]	0.002 [0.001, 0.003]	0.002 [0.001, 0.004]
Percent of days Low Staffing: CNA	0.018 [0.008, 0.027]	0.007 [0.000, 0.013]	0.003 [-0.005, 0.012]	0.012 [0.004, 0.020]	0.017 [0.006, 0.029]	0.006 [0.002, 0.010]	0.030 [0.008, 0.053]	-0.004 [-0.016, 0.009]	0.006 [-0.005, 0.017]	0.003 [0.001, 0.004]	0.004 [0.003, 0.006]	0.006 [0.004, 0.008]
Average HPRD: RN	-0.826 [-1.213, -0.440]	0.075 [-0.226, 0.375]	0.050 [-0.327, 0.426]	-0.698 [-1.094, -0.303]	-0.303 [-0.802, 0.196]	-0.210 [-0.301, -0.118]	1.422 [0.653, 2.190]	-0.534 [-0.865, -0.204]	-1.113 [-1.378, -0.849]	-0.186 [-0.245, -0.127]	-0.174 [-0.225, -0.122]	-0.278 [-0.323, -0.234]
Average HPRD: LPN	0.889 [0.565, 1.214]	0.529 [0.270, 0.787]	1.141 [0.773, 1.509]	-0.387 [-0.726, -0.049]	1.206 [0.788, 1.623]	0.031 [-0.065, 0.127]	1.359 [0.586, 2.132]	0.991 [0.613, 1.368]	0.714 [0.386, 1.043]	0.067 [0.014, 0.121]	0.058 [0.012, 0.103]	-0.005 [-0.053, 0.043]
Average HPRD: CNA	-0.062 [-0.247, 0.123]	-0.155 [-0.274, -0.037]	-0.396 [-0.565, -0.227]	0.116 [-0.069, 0.301]	-0.261 [-0.482, -0.041]	-0.007 [-0.064, 0.051]	-0.996 [-1.431, -0.562]	-0.253 [-0.473, -0.033]	-0.110 [-0.290, 0.070]	0.045 [0.016, 0.073]	0.031 [0.006, 0.055]	-0.047 [-0.076, -0.018]
Case-mix index	-1.428 [-1.797, -1.059]	1.167 [0.886, 1.448]	1.636 [1.239, 2.033]	-3.217 [-3.639, -2.796]	0.834 [0.366, 1.302]	-0.273 [-0.380, -0.166]	-0.676 [-1.559, 0.208]	0.038 [-0.333, 0.409]	-0.835 [-1.137, -0.533]	0.058 [0.008, 0.108]	-0.061 [-0.106, -0.017]	-0.034 [-0.087, 0.019]
% Residents below age 65	-0.002 [-0.011, 0.008]	0.044 [0.037, 0.052]	0.040 [0.031, 0.050]	0.127 [0.113, 0.141]	-0.003 [-0.015, 0.009]	0.018 [0.014, 0.021]	0.053 [0.025, 0.081]	0.028 [0.017, 0.039]	0.019 [0.009, 0.028]	0.003 [0.002, 0.004]	0.007 [0.005, 0.008]	0.008 [0.006, 0.010]
% Residents age 65-74	0.027 [0.015, 0.040]	0.044 [0.035, 0.053]	0.054 [0.042, 0.066]	0.078 [0.063, 0.092]	0.030 [0.015, 0.045]	0.002 [-0.002, 0.006]	0.042 [0.010, 0.074]	0.005 [-0.009, 0.020]	0.010 [-0.003, 0.022]	0.007 [0.005, 0.009]	0.005 [0.004, 0.007]	0.008 [0.006, 0.010]
% Residents age 75 to 84	0.023 [0.011, 0.034]	0.008 [-0.000, 0.016]	0.018 [0.007, 0.029]	0.057 [0.044, 0.070]	0.022 [0.008, 0.036]	0.006 [0.002, 0.010]	0.058 [0.029, 0.087]	0.036 [0.021, 0.050]	0.015 [0.003, 0.028]	0.003 [0.001, 0.004]	0.003 [0.001, 0.004]	0.003 [0.001, 0.005]

© 2023 Mukamel DB et al. *JAMA Network Open.*

**eTable.** Full Regression Models												
	**Long-stay**					**Short-stay**				**Long-stay**		
	**ADL increase**	**High-risk pressure ulcer FYs Q1 2017-Q3 2018**	**High-risk pressure ulcer FYs Q4 2018-Q3 2019**	**Anti-psychotic medi-cation**	**Mobility decline**	**Anti-psychotic medi-cation**	**Mobility did not decline**	**Re-hospitali zation**	**ED visit**	**Number of hospitali zation**	**ED visit**	**Deficien-cies: Z Score**
	**Coefficients [95% CI]**											
% Residents age 85 and above (ref)	----	----	----	----	----	----	----	----	----	----	----	----
% Male residents	-0.003 [-0.012, 0.005]	0.003 [-0.003, 0.009]	0.007 [-0.001, 0.015]	0.031 [0.019, 0.043]	-0.023 [-0.034, -0.012]	0.011 [0.007, 0.015]	0.006 [-0.019, 0.032]	-0.017 [-0.028, -0.006]	-0.009 [-0.018, 0.001]	-0.001 [-0.002, 0.000]	-0.005 [-0.006, -0.004]	-0.000 [-0.002, 0.001]
Resident Census	-0.003 [-0.005, -0.001]	-0.001 [-0.002, -0.000]	-0.000 [-0.002, 0.001]	0.003 [0.000, 0.006]	-0.006 [-0.009, -0.004]	0.001 [0.000, 0.002]	0.003 [-0.001, 0.008]	-0.001 [-0.003, 0.000]	-0.006 [-0.007, -0.004]	0.000 [-0.000, 0.000]	-0.001 [-0.001, -0.001]	0.002 [0.002, 0.002]
% Residents with payer: Medicare	-0.014 [-0.024, -0.003]	0.005 [-0.002, 0.011]	0.021 [0.010, 0.032]	-0.003 [-0.016, 0.010]	-0.011 [-0.024, 0.002]	-0.002 [-0.004, 0.001]	-0.02 [-0.043, 0.002]	0.023 [0.014, 0.032]	0.008 [0.000, 0.015]	0.000 [-0.001, 0.002]	0.001 [-0.000, 0.002]	-0.000 [-0.002, 0.001]
% Residents with payer: Other	-0.009 [-0.014, -0.004]	0.004 [0.001, 0.008]	0.007 [0.002, 0.011]	-0.003 [-0.010, 0.004]	-0.005 [-0.011, 0.001]	-0.004 [-0.005, -0.002]	-0.007 [-0.020, 0.006]	-0.003 [-0.008, 0.003]	-0.005 [-0.010, -0.001]	-0.003 [-0.003, -0.002]	-0.002 [-0.003, -0.001]	0.000 [-0.001, 0.001]
% Residents with payer: Medicaid (ref)	----	----	----	----	----	----	----	----	----	----	----	----
Nursing Home Occupancy	-0.024 [-0.030, -0.017]	-0.013 [-0.017, -0.009]	-0.020 [-0.026, -0.014]	0.004 [-0.007, 0.014]	-0.023 [-0.031, -0.014]	0.001 [-0.001, 0.003]	-0.001 [-0.017, 0.016]	-0.009 [-0.016, -0.002]	-0.013 [-0.019, -0.007]	-0.002 [-0.002, -0.001]	-0.003 [-0.004, -0.002]	-0.005 [-0.006, -0.004]
Nursing Home is for profit	0.155 [-0.053, 0.362]	-0.028 [-0.163, 0.107]	0.067 [-0.112, 0.247]	-0.096 [-0.437, 0.245]	-0.185 [-0.451, 0.082]	0.044 [-0.014, 0.101]	-0.149 [-0.647, 0.350]	0.270 [0.070, 0.470]	0.150 [-0.025, 0.326]	0.044 [0.019, 0.069]	0.042 [0.018, 0.067]	0.084 [0.053, 0.115]
Nursing home is part of a chain	0.303 [0.135, 0.471]	-0.107 [-0.214, 0.001]	-0.098 [-0.247, 0.051]	-0.243 [-0.506, 0.020]	0.461 [0.247, 0.674]	-0.045 [-0.096, 0.006]	0.409 [-0.016, 0.835]	-0.037 [-0.200, 0.125]	0.316 [0.173, 0.459]	-0.057 [-0.077, -0.037]	0.025 [0.006, 0.045]	-0.004 [-0.032, 0.024]
Nursing home is	0.512 [0.021, 1.002]	0.589 [0.175, 1.003]	0.240 [-0.285, 0.765]	-0.857 [-1.602, -0.111]	0.703 [0.043, 1.363]	-0.289 [-0.420, -0.157]	2.321 [1.043, 3.598]	-1.515 [-2.074, -0.957]	-0.217 [-0.707, 0.273]	-0.072 [-0.145, 0.000]	0.067 [-0.009, 0.143]	0.016 [-0.046, 0.078]

© 2023 Mukamel DB et al. *JAMA Network Open.*

**eTable.** Full Regression Models												
	**Long-stay**					**Short-stay**				**Long-stay**		
	**ADL increase**	**High-risk pressure ulcer FYs Q1 2017-Q3 2018**	**High-risk pressure ulcer FYs Q4 2018-Q3 2019**	**Anti-psychotic medi-cation**	**Mobility decline**	**Anti-psychotic medi-cation**	**Mobility did not decline**	**Re-hospitali zation**	**ED visit**	**Number of hospitali zation**	**ED visit**	**Deficien-cies: Z Score**
	**Coefficients [95% CI]**											
hospital-based												
% county population in rural areas	-0.005 [-0.009, -0.000]	-0.010 [-0.013, -0.007]	-0.011 [-0.015, -0.007]	0.026 [0.018, 0.033]	-0.008 [-0.014, -0.003]	0.001 [-0.001, 0.002]	-0.057 [-0.069, -0.044]	-0.021 [-0.026, -0.016]	0.017 [0.012, 0.021]	-0.001 [-0.002, -0.001]	0.003 [0.003, 0.004]	-0.002 [-0.003, -0.001]
County HHI	0.727 [0.192, 1.261]	0.499 [0.135, 0.862]	0.569 [0.103, 1.036]	-0.679 [-1.507, 0.149]	0.094 [-0.559, 0.747]	-0.055 [-0.244, 0.134]	-0.451 [-1.985, 1.083]	-0.148 [-0.720, 0.424]	1.482 [0.928, 2.036]	0.083 [0.019, 0.146]	0.423 [0.344, 0.502]	0.037 [-0.041, 0.115]
Time interval 1* (ref)	----	----		----	----	----	----	----	----	----		----
Time interval 2	-0.754 [-0.908, -0.601]	-0.171 [-0.241, -0.100]		-0.209 [-0.283, -0.135]	-1.159 [-1.342, -0.975]	-0.072 [-0.126, -0.018]	0.013 [-0.246, 0.271]	0.338 [0.203, 0.474]	-1.680 [-1.792, -1.568]	0.020 [0.007, 0.034]	----	0.052 [0.022, 0.081]
Time interval 3	-0.939 [-1.102, -0.776]	-0.317 [-0.396, -0.238]		-0.306 [-0.397, -0.216]	-1.675 [-1.866, -1.484]	-0.140 [-0.194, -0.086]	-0.765 [-1.049, -0.482]	-1.133 [-1.268, -0.998]	-2.109 [-2.221, -1.997]	-0.008 [-0.022, 0.007]	-0.062 [-0.071, -0.053]	0.039 [0.010, 0.068]
Time interval 4	-0.493 [-0.660, -0.326]	-0.264 [-0.347, -0.181]		-0.532 [-0.634, -0.429]	-1.014 [-1.211, -0.817]	-0.174 [-0.230, -0.119]	0.525 [0.214, 0.837]					
Time interval 5	0.064 [-0.105, 0.234]	-0.151 [-0.238, -0.064]		-0.795 [-0.909, -0.680]	0.071 [-0.134, 0.277]	-0.210 [-0.264, -0.155]	-0.112 [-0.435, 0.210]					
Time interval 6	-0.992 [-1.164, -0.821]	-0.280 [-0.368, -0.191]		-1.150 [-1.272, -1.027]	-1.440 [-1.645, -1.234]	-0.213 [-0.269, -0.158]	-1.064 [-1.403, -0.724]					
Time interval 7	-1.155 [-1.326, -0.984]	-0.446 [-0.535, -0.358]		-1.194 [-1.323, -1.066]	-1.276 [-1.484, -1.068]							

© 2023 Mukamel DB et al. *JAMA Network Open.*

**eTable.** Full Regression Models												
	**Long-stay**					**Short-stay**				**Long-stay**		
	**ADL increase**	**High-risk pressure ulcer FYs Q1 2017-Q3 2018**	**High-risk pressure ulcer FYs Q4 2018-Q3 2019**	**Anti-psychotic medi-cation**	**Mobility decline**	**Anti-psychotic medi-cation**	**Mobility did not decline**	**Re-hospitali zation**	**ED visit**	**Number of hospitali zation**	**ED visit**	**Deficien-cies: Z Score**
	**Coefficients [95% CI]**											
Time interval 8 (ref#)	-0.853 [-1.023, -0.683]		----	-1.211 [-1.344, -1.078]	-1.157 [-1.369, -0.946]							
Time interval 9	-0.683 [-0.856, -0.509]		0.158 [0.083, 0.233]	-1.243 [-1.380, -1.107]	-1.398 [-1.608, -1.188]							
Time interval 10	-1.355 [-1.529, -1.181]		0.060 [-0.026, 0.147]	-1.500 [-1.641, -1.358]	-2.210 [-2.420, -2.000]							
Time interval 11	-1.307 [-1.483, -1.131]		-0.035 [-0.126, 0.057]	-1.712 [-1.859, -1.564]	-2.370 [-2.581, -2.159]							
State Fixed Effects**	----	----	----	----	----	----	----	----	----	----	----	----
Constant	16.119 [14.801, 17.438]	3.361 [2.485, 4.238]	3.964 [2.744, 5.185]	17.209 [15.436, 18.981]	16.915 [15.253, 18.578]	1.572 [1.190, 1.954]	-63.875 [-67.147, -60.603]	22.458 [21.143, 23.773]	12.513 [11.390, 13.635]	1.718 [1.537, 1.900]	0.786 [0.634, 0.938]	-0.060 [-0.259, 0.139]
Number of observations	138,375	83,444	48,994	140,849	132,424	58,223	47,685	33,571	33,571	34,046	23,539	28,722
Number of nursing homes	13,962	13,427	12,973	14,116	13,671	11,815	10,019	13,196	13,196	13,299	12,854	13,999
R-squared	0.092	0.109	0.118	0.187	0.071	0.056	0.091	0.102	0.140	0.165	0.263	0.069

* Time intervals vary by outcome. ADLs, pressure sores, long-stay mobility, and long-stay antipsychotic medication are measured quarterly. Short-stay movement and short-stay antipsychotic medication and deficiencies are measured over 6 months. The time intervals for short and long-stay ED and hospitalizations are annual.

** Coefficients and p-values for state fixed effects omitted.

© 2023 Mukamel DB et al. *JAMA Network Open.*
