# Supplementary material for: Association of Staffing Instability With Quality of Nursing Home Care
Source: JAMA Netw Open. 2023 Jan 10;6(1):e2250389. doi: 10.1001/jamanetworkopen.2022.50389 (PMC9856742; doi:10.1001/jamanetworkopen.2022.50389)
Supplement: Supplement 2. — Data Sharing Statement [file jamanetwopen-e2250389-s002.pdf]

Data Sharing Statement

Mukamel. Association of Staffing Instability With Quality of Nursing Home Care. *JAMA Netw Open*. Published January 10, 2023. doi:10.1001/jamanetworkopen.2022.50389

Data
Data available: No

Additional Information
Explanation for why data not available: We are unable to share the data because data were obtained from CMS under a DUA. Those interested in obtaining the code should contact the PI.
